# Diagnostic Limitation of Fine-Needle Aspiration (FNA) on Indeterminate Thyroid Nodules Can Be Partially Overcome by Preoperative Molecular Analysis: Assessment of *RET/PTC1* Rearrangement in *BRAF* and *RAS* Wild-Type Routine Air-Dried FNA Specimens

**DOI:** 10.3390/ijms18040806

**Published:** 2017-04-12

**Authors:** Young Sin Ko, Tae Sook Hwang, Ja Yeon Kim, Yoon-La Choi, Seung Eun Lee, Hye Seung Han, Wan Seop Kim, Suk Kyeong Kim, Kyoung Sik Park

**Affiliations:** 1 Diagnostic Pathology Center, Seegene Medical Foundation, Seoul KS013, Korea; noteasy@mf.seegene.com; 2 Molecular Genetics and Pathology, Department of Medicine, Graduate School of Konkuk University, Seoul KS013, Korea; aphsh@kuh.ac.kr (H.S.H.); wskim@kuh.ac.kr (W.S.K.); 3 Department of Pathology, Konkuk University School of Medicine, Seoul KS013, Korea; 78jykim@hanmail.net; 4 Department of Pathology and Translational Genomics, Samsung Medical Center, Sungkyunkwan University School of Medicine, Seoul KS013, Korea; yla.choi@samsung.com; 5 Department of Pathology, Konkuk University Medical Center, Seoul KS013, Korea; 20150063@kuh.ac.kr; 6 Department of Internal Medicine, Konkuk University School of Medicine, Seoul KS013, Korea; endolife@kuh.ac.kr; 7 Department of Surgery, Konkuk University School of Medicine, Seoul KS013, Korea; kspark@kuh.ac.kr

**Keywords:** *RET/PTC* gene rearrangement, air-dried FNA specimen, RT-PCR, Nanostring

## Abstract

Molecular markers are helpful diagnostic tools, particularly for cytologically indeterminate thyroid nodules. Preoperative *RET/PTC1* rearrangement analysis in *BRAF* and *RAS* wild-type indeterminate thyroid nodules would permit the formulation of an unambiguous surgical plan. Cycle threshold values according to the cell count for detection of the *RET/PTC1* rearrangement by real-time reverse transcription-polymerase chain reaction (RT-PCR) using fresh and routine air-dried TPC1 cells were evaluated. The correlation of *RET/PTC1* rearrangement between fine-needle aspiration (FNA) and paired formalin-fixed paraffin-embedded (FFPE) specimens was analyzed. *RET/PTC1* rearrangements of 76 resected *BRAF* and *RAS* wild-type classical PTCs were also analyzed. Results of RT-PCR and the Nanostring were compared. When 100 fresh and air-dried TPC1 cells were used, expression of *RET/PTC1* rearrangement was detectable after 35 and 33 PCR cycles, respectively. The results of *RET/PTC1* rearrangement in 10 FNA and paired FFPE papillary thyroid carcinoma (PTC) specimens showed complete correlation. Twenty-nine (38.2%) of 76 *BRAF* and *RAS* wild-type classical PTCs had *RET/PTC1* rearrangement. Comparison of *RET/PTC1* rearrangement analysis between RT-PCR and the Nanostring showed moderate agreement with a κ value of 0.56 (*p* = 0.002). The *RET/PTC1* rearrangement analysis by RT-PCR using routine air-dried FNA specimen was confirmed to be technically applicable. A significant proportion (38.2%) of the *BRAF* and *RAS* wild-type PTCs harbored *RET/PTC1* rearrangements.

## 1. Introduction

The evaluation of a thyroid nodule is a very common clinical problem. Epidemiologic studies have shown the prevalence of palpable thyroid nodules to be approximately 5% in women and 1% in men living in iodine-sufficient parts of the world [1,2]. In contrast, high-resolution ultrasound (US) can detect thyroid nodules in 19–68% of randomly selected individuals, with higher frequencies in women and the elderly [3,4]. The clinical importance of thyroid nodules rests with the need to exclude thyroid cancer, which occurs in 7–15% of cases depending on age, sex, radiation exposure history, family history, and other factors [5,6]. Differentiated thyroid cancer (DTC) includes papillary and follicular cancer, and comprises the vast majority (90%) of all thyroid cancers [7]. In the United States, approximately 63,000 new cases of thyroid cancer were predicted to be diagnosed in 2014 [8] compared with 37,200 in 2009 when the last ATA guidelines were published. The yearly incidence has nearly tripled from 4.9 per 100,000 in 1975 to 14.3 per 100,000 in 2009 [9].

The most prevalent type of thyroid malignancy in Korea is papillary thyroid carcinoma (PTC), which constitutes more than 97% of the cases, followed by follicular thyroid carcinoma (FTC), comprising 1.5% of the thyroid cancer [10]. Compared to Western countries, the prevalence of PTC is much higher. Therefore, the evaluation of a thyroid nodule in Korea is primarily a search for PTC.

Fine-needle aspiration (FNA) is the safest and most reliable test that can provide a definitive preoperative diagnosis of malignancy [11]. The sensitivity and specificity of FNA are reported to be 68–98% and 56–100%, respectively [12]. However, 15–30% of thyroid FNA diagnoses are “atypia of undetermined significance (AUS)/follicular lesion of undetermined significance (FLUS)”, “follicular neoplasm or suspicious for follicular neoplasm (FN/SFN)”, and “suspicious for malignancy” [13]. This leads to an increased rate of unnecessary surgery, as only about 25% of the indeterminate cases will receive a postoperative malignant diagnosis by histological examination [11]. Moreover, patients with a diagnosis of indeterminate category usually undergo hemithyroidectomy, and about 25% of the patients need to have a second stage completion thyroidectomy in most centers [12]. Two-stage surgery has higher morbidity than initial total thyroidectomy undertaken with a definitive malignant diagnosis on FNA. Preoperative molecular analysis using a panel of genetic alterations would overcome the limitation of FNA diagnosis. The most common genetic alteration in thyroid cancer is the activation of the mitogen-activated protein kinase pathway. Activation of this pathway occurs through mutually exclusive mutations of the *BRAF* and *RAS* genes and rearrangements of the *RET/PTC* and *NTRK*. The overall prevalence of the *BRAF* mutations is approximately 45% (range, 27.3–87.1%) [14,15], with a significantly higher prevalence in Asia—especially Korea—relative to Western countries [15,16,17]. The mutations of the *RAS* genes are the second most common genetic alterations in thyroid tumors, and are mostly present in follicular-patterned lesions. The prevalence of *RAS* mutations in follicular variant of papillary thyroid carcinoma (FVPTC) varies from 26.5% to 33.3% in Korea, where most of the follicular patterned thyroid malignancy is FVPTC [18,19].

*RET* proto-oncogene rearrangements are commonly seen in PTC. These rearrangements play a role in pathogenesis of PTC, and derive from the fusion of the *RET* tyrosine kinase domain sequence with 50 sequences of heterologous genes. The resulting chimeric oncogenes are termed *RET/PTCs* [20,21,22,23,24]. *RET/PTC* rearrangements are typically common in tumors from patients with a history of radiation exposure (50–80%) and PTC of children and young adults (40–70%) [25,26]. The distribution of *RET/PTC* rearrangements within this tumor is quite heterogeneous, and varies from the involvement of almost all neoplastic cells to presence in only a small fraction of the tumor cells [27,28]. To date, 13 different types of *RET/PTC* rearrangements have been reported; *RET/PTC1* and *RET/PTC3* account for more than 90% of all rearrangements.

The prevalence of the *RET/PTC* rearrangements in PTC varies widely in different populations (range, 0–86.8%) [29,30,31], with significant variability in mutational frequency—even within the same geographical regions. Rates of 0–54.5% have been reported in Asia [30,31,32], 2.4–72.0% in the United States [17,33], and 8.1–42.9% in Europe [34,35]. The marked variations may reflect the small size of the studies, geographic variability, or different sensitivities of the detection methods [36,37]. When this variability is considered, the prevalence of *RET/PTC* rearrangements in Asia is generally low [29,30,31,32,38,39]. The subclonal occurrence of *RET/PTC* rearrangement in PTC can influence the sensitivity of some methods, and might explain why the reported prevalence of *RET/PTC* rearrangements in PTCs varies in different studies. Very recent studies demonstrated that *RET/PTC* rearrangements in benign thyroid nodules are not an uncommon occurrence, and suggested that its presence could be associated with a faster nodular enlargement [40,41,42]. A variety of methods have been used to identify *RET/PTC* rearrangements. These include real-time reverse transcription-polymerase chain reaction (RT-PCR), Southern blot analysis, fluorescence in situ hybridization, and NanoString nCounter Gene Expression Assay.

Most preoperative detection of these rearrangements has been performed in fresh FNA material. Recently, detection of the *PAX8/PPARG* and *RET/PTC* rearrangements in routine air-dried FNA samples was reported [43,44,45,46,47,48]. The FNA approach suffers from the limitation that indeterminate FNA specimens usually contain small numbers of atypical cells, and these cells are often mixed with many inflammatory cells, benign follicular cells, and stromal cells. Therefore, harvesting the cells of interest is the key step in molecular analysis of the FNA specimen.

Preoperative *RET/PTC1* rearrangement analysis in *BRAF* and *RAS* wild-type indeterminate thyroid nodules would permit the formulation of an unambiguous surgical plan, while foregoing the need for other less-specific diagnostic tests like repeat FNA and intraoperative frozen section evaluation. We have previously reported the value of the preoperative *BRAF* and *RAS* mutation analysis in diagnosing PTC in routine air-dried FNA specimens [18,49,50,51]. In our institution, we recommend surgery for *BRAF* or *RAS*-positive thyroid nodules with preoperative cytological diagnosis of AUS/FLUS and FN/SFN categories, and have been able to detect considerable numbers of PTCs in cytologically-indeterminate nodules [50]. Considering that 88% of the PTCs harbor either a *BRAF* or a *RAS* mutation (Thyroid, 2017, Epub ahead of time), we hypothesized that detection of *RET/PTC* rearrangements on *BRAF* and *RAS* mutation wild-type FNA specimens of the indeterminate thyroid nodules will improve the diagnostic yield of PTC. An algorithmic approach is cost-effective and efficient—especially in *BRAF* mutation-prevalent populations.

In this study, we investigated the clinical feasibility of preoperative *RET/PTC1* rearrangement analysis as an ancillary diagnostic tool in routine air-dried FNA samples. We also evaluated the *RET/PTC1* rearrangement status for 76 *BRAF* and *RAS* wild-type classical PTC cases.

## 2. Results

### 2.1. Detection of the RET/PTC1 Rearrangement in a Fresh TPC1 Cell Line

**Figure 1:**
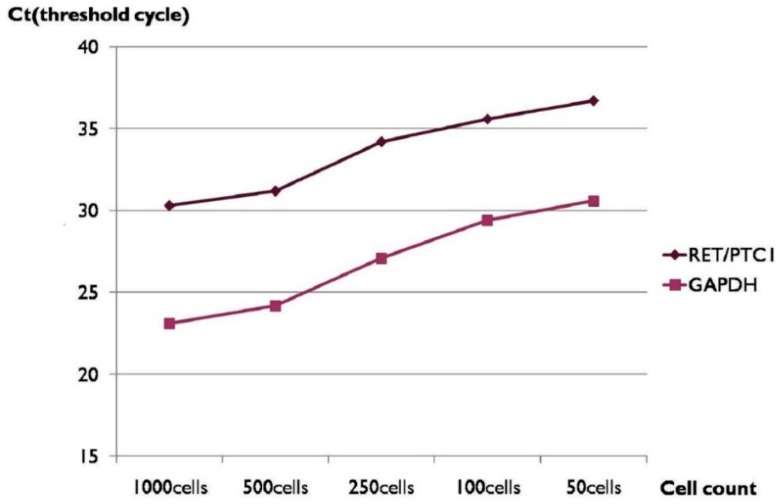
Threshold cycle (*C*_t_) values and cell counts of *RET/PTC1* rearrangement analysis by real-time reverse transcription-polymerase chain reaction (RT-PCR) using fresh cultured TPC1 cells. *GAPDH*: glyceraldehyde-3-phosphate dehydrogenase.

The *C*_t_ value was increased when the cell numbers used for analysis were decreased and showed an inverse correlation (Table 1 and Figure 1). *RET/PTC1* rearrangement was detectable after 35 PCR cycles when 100 TPC1 cells were used.

### 2.2. Detection of the RET/PTC1 Rearrangement in Routine Air-Dried TPC1 Cell Line

**Figure 2:**
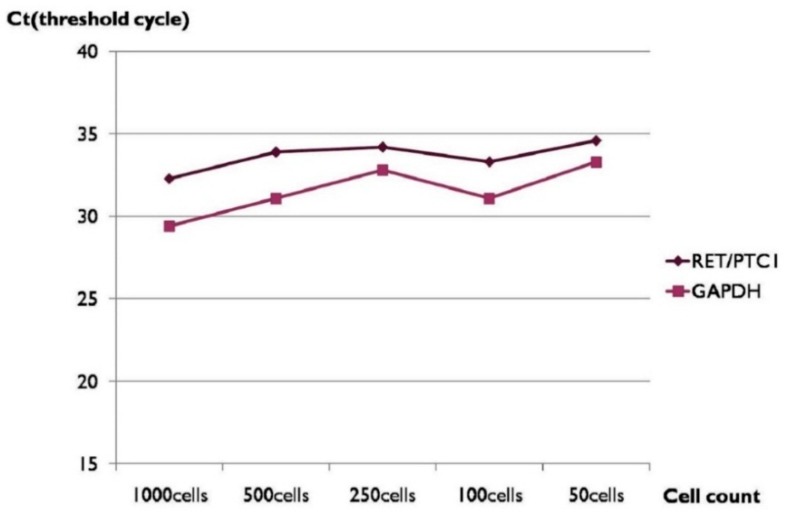
*C*_t_ values and cell counts of *RET/PTC1* rearrangement analysis by RT-PCR using RNA extracted from routine air-dried and Papanicolaou-stained TPC1 cells.

When *RET/PTC1* rearrangement was analyzed using various numbers of smeared, alcohol-fixed, and Papanicolaou-stained PTC1 cells, the cell number and the threshold cycle (*C*_t_) value also showed an inverse correlation (Table 2 and Figure 2). The expression of *RET/PTC1* rearrangement was detectable after 33 PCR cycles when 100 cells were used.

### 2.3. Correlation of the RET/PTC1 Rearrangement between Routine Air-Dried FNA and Paired FFPE PTC Tissue Specimens

When *RET/PTC1* rearrangement was analyzed using PTC cells from archival air-dried FNA slides aspirated from the patients proven to have a histopathological diagnosis of PTC, *RET/PTC1* rearrangement was detected in all 6 cases even though the *C*_t_ values of the archival specimen were higher than those of formalin-fixed paraffin-embedded (FFPE) PTC tissue specimens (Table 3). Four cases lacking *RET/PTC1* rearrangement in tissue specimen also failed to reveal rearrangement in FNA samples. These results confirmed that *RET/PTC1* rearrangement analysis by RT-PCR can be applied in preoperative FNA samples as an ancillary diagnostic tool.

### 2.4. Detection of the RET/PTC1 Rearrangement in Resected BRAF and RAS Wild-Type PTC Cases Using FFPE Tissue Specimen

Of 600 surgically resected FFPE specimens histologically diagnosed as PTC, classical type, 518 had *BRAF* mutations and 6 had *RAS* mutations. Among 76 *BRAF* and *RAS* wild-type PTCs, 29 (38.2%) cases turned out to have *RET/PTC1* rearrangement. Considering that alteration of *BRAF*, *RAS*, and *RET* genes are mutually exclusive, 29 (4.8%) of 600 classical PTC cases harbored *RET/PTC1* rearrangement.

### 2.5. Comparative Analysis of RT-PCR with the NanoString nCounter Gene Expression Assay for Detecting RET/PTC1 Rearrangement in FFPE PTC Tissue Specimen

Twenty-six cases showed correlation on both methods (5 positives and 21 negatives), whereas five cases showed discrepancy between the two methods (three cases positive for Nanostring but not for RT-PCR, two cases positive for RT-PCR but not for Nanostring). Two different analysis methods showed moderate agreement with a κ value of 0.56 (*p* = 0.002).

## 3. Discussion

The value of molecular markers on preoperative FNA specimens has been described in various thyroid nodules [18,47,49,50,51,52,53]. *RET/PTC* rearrangements are commonly found in adult sporadic PTCs with a marked variable prevalence in different studies owing to geographic variability or different sensitivity of the detection methods [17,29,30,31,32,33,34,35,36,37]. The reported prevalence rates of *RET/PTC* rearrangements varied largely among studies. While geographical factors and radiation exposure can partially account for this wide range of prevalence, the methodology applied appears to be the most important factor to explain this variability. Searching for *RET/PTC* rearrangements by a less sensitive method may have the drawback of leaving some PTCs undiagnosed, but has the advantage of reducing false positive findings. Indeed, while sporadic cells harboring *RET/PTC* rearrangements can be present in benign nodules, its clonal occurrence is exclusive to PTC. Hence, the less sensitive RT-PCR seems to be more suitable for diagnostic purposes. *RET/PTC* rearrangements analysis on thyroid tumor has not been extensively performed in Korea, given the prevalence of the *BRAF* V600E mutation in PTC in Korea.

In this report, we assessed the clinical usability of preoperative *RET/PTC1* rearrangement analysis as an ancillary diagnostic tool for *BRAF* and *RAS* wild-type indeterminate thyroid nodules, and explored the *RET/PTC1* rearrangement status in a large number of PTC cases. These explorations have never been done in Korea, to our knowledge. We routinely use atypical follicular cells marked by the cytopathologists and dissected from routine air-dried FNA samples to increase the sensitivity. Since clinical FNA samples contain limited numbers of cells to perform several steps required for deciding optimum number of cells for successful analysis and cutoff values, we performed same analysis using fresh TPC1 cells which are equivalent to the fresh FNA samples in step 1 and air-dried Papanicolaou-stained TPC1 cells equivalent to the archival FNA slides in step 2.

The *C*_t_ values in Table 2 tend to decrease when the cell numbers were increased; however, both *C*_t_ values of the *RET/PTC1* and *GAPDH* expression using 250 cells are greater than those using 100 cells. Since *C*_t_ values of the housekeeping gene expression also showed the same phenomenon, we assumed that a considerable amount of RNA in 250-cell groups might have been deteriorated. At any rate, we found that *RET/PTC1* expression could be measured when 50–100 air-dried Papanicolaou-stained TPC1 cells were used.

When we compared the *C*_t_ values of fresh and air-dried Papanicolaou-stained TPC1 cells according to the cell numbers, the *C*_t_ values were slightly decreased when air-dried Papanicolaou-stained cells were used. We assumed that this finding might have resulted from the imprecise cell count in step 2. Fresh cells were counted using a hemocytometer, whereas air-dried and Papanicolaou-stained cells were counted on a slide using a square micrometer under the microscope. The expression of *RET/PTC1* rearrangement detectable after 33 PCR cycles when routine air-dried 100 TPC1 cells were used suggests that *RET/PTC1* expression could be detected in routine air-dried FNA samples containing 100 cells.

When *RET/PTC1* rearrangement status from ten FNA and paired FFPE samples were compared, the results showed complete agreement. The higher *C*_t_ value of FNA samples compared to the matched FFPE samples could be attributed to the much smaller numbers of cells in FNA samples. The other reason might be the different RNA extraction method used for the two different samples.

Since *BRAF* mutations, *RAS* mutations, and *RET/PTC* rearrangements are mutually exclusive, we analyzed the *RET/PTC1* rearrangement status on both *BRAF* (V600E and K601E) and *RAS* (*NRAS* codons 12, 13, 61; *HRAS* codons 12, 13, 61; *KRAS* codons 12, 13, 61) wild-type PTC cases to save cost and effort. The main limitation of our experiment is that we only performed *RET/PTC1* rearrangement, even though the prevalence for *RET/PTC3* arrangement in previous Korean report was 0%. Another reason for analyzing *RET/PTC1* is that we were able to secure a cell line harboring only the *RET/PTC1* rearrangement.

Among 76 surgically resected both *BRAF* and *RAS* wild-type FFPE specimens histopathologically diagnosed as PTC, classical type, 29 (38.2%) cases turned out to have *RET/PTC1* rearrangement; this means that *RET/PTC1* rearrangement was detected in 29 (4.8%) of 600 classical-type PTCs. Two previous studies reported *REP/PTC* rearrangements in Korea. One study failed to identify any *RET/PTC1*, *2*, *3* rearrangements in 24 cases of PTC by RT-PCR [31]. The other study detected 2 (6.5%) *RET/PTC1*, 2 (6.5%) *RET/PTC2*, and no (0%) *RET/PTC3* rearrangements in 31 PTCs by RT-PCR [38]. Both studies used fresh frozen tumor tissue. The slight discrepancy could be explained by the difference of the sample size. The slightly lower prevalence of *RET/PTC1* rearrangement compared to the second study might also be attributed to the poor RNA preservation in the FFPE specimens.

The NanoString nCounter Gene Expression Assay is a robust and highly reproducible method for detecting the expression of up to 800 genes in a single reaction with high sensitivity and linearity across a broad range of expression levels. The methodology serves to bridge the gap between genome-wide (microarrays) and targeted (real-time quantitative PCR) expression profiling. The nCounter assay is based on direct digital detection of mRNA molecules of interest using target-specific, color-coded probe pairs. It does not require the conversion of mRNA to cDNA by reverse transcription or the amplification of the resulting cDNA by PCR. The expression level of a gene is measured by counting the number of times the color-coded barcode for that gene is detected, and the barcode counts are then tabulated [54]. Comparative analysis of RT-PCR with the Nanostring method for detecting *RET/PTC1* rearrangement in FFPE PTC tissue showed moderate agreement with a *k* value of 0.56 (*p* = 0.002). There is a discrepancy between these two methods (three cases positive for Nanostring but not for RT-PCR, two cases positive for RT-PCR but not for Nanostring). The discrepancy might be attributed to the different RNA extraction methods and cut-off values of each method. In three cases with discrepancy, the results were near to the cut-off value. Another reason might be attributed to the difference of tumor portion used in two different methods. Since we did not initially plan to compare RT-PCR with Nanostring, we made the tumor sections only for RT-PCR analysis. Therefore, the tumor portions which were used for Nanostring might have been slightly different from the initial tumor portion. Next generation sequencing (NGS) is being used to study genetic alterations in institutions worldwide. However, it may be a long time until NGS becomes a routine part of thyroid cancer practice in Korea, since only NGS panels relevant for the therapeutic modality have been approved by the Korean government. Furthermore, only large institutions like university hospitals can adopt NGS in practice. Therefore, algorithmic approach of *BRAF* mutation analysis followed by *RAS* mutation and *RET/PTC1* rearrangement may be of more practical help to refine FNA diagnosis of indeterminate thyroid nodules.

We confirmed the technical applicability of *RET/PTC1* rearrangement analysis using routine air-dried FNA samples as an ancillary diagnostic tool through several steps of the experiment. The presence of *RET/TPC1* rearrangement in a significant proportion (38.2%) of the patients with *BRAF* and *RAS* wild-type PTCs can be used to diagnose and manage patients with *BRAF* and *RAS* wild-type indeterminate thyroid nodules. Since the *BRAF* V600E mutation, *NRAS* codon 61 mutation, and *RET/PTC1* rearrangement comprise more than 90%, 75%, and 50% of the *BRAF* mutations, *RAS* mutations, and *RET/PTC* rearrangements [18,50], an algorithmic approach of *BRAF* V600E mutation analysis followed by *NRAS* 61 mutation and *RET/PTC1* rearrangement analysis would cost-effectively and efficiently overcome a diagnostic limitation of the thyroid FNA by triaging considerable numbers of PTCs in cytologically indeterminate nodules.

## 4. Materials and Methods

### 4.1. Total RNA Extraction and First-Strand Synthesis

Total RNA from fresh and fixed TPC1 cells (derived from human thyroid papillary carcinoma, classic type and harboring *RET/PTC1* rearrangement) was extracted using MasterPure Complete DNA and RNA Purification Kit (Epicentre, Madison, WI, USA). Total RNA from formalin-fixed paraffin-embedded (FFPE) specimen was extracted using a High Pure FFPE RNA isolation kit (Roche Diagnostics, Mannheim, Germany). First-strand synthesis was performed on 2 µg of total RNA using a Tetro cDNA synthesis kit (Bioline, London, UK). Tetro reverse transcriptase with diethyl pyrocarbonate water and cDNA reverse transcribed product from the TPC1 cells were used as negative and positive controls, respectively.

### 4.2. RT-PCR

Amplification was performed by RT-PCR using a LightCycler 480 Instrument (Roche Diagnostics), and measurement was performed using LightCycler quantification software version 1.5 (Roche Diagnostics). The RT-PCR reaction mixture was prepared in a Light Cycler^®^ 480 Multiwell Plate 96 containing 0.5 μM of each primer set (*RET/PTC1* and *glyceraldehyde-3-phosphate isomerase, GAPDH*), 0.25 μM of the probes, 2X of LightCycler 480 Probes Master (Roche Diagnostics), and 1–2 μg (1 µg for the cell and 2 µg for the FFPE tissue) of cDNA template in a final reaction volume of 20 μL (Table 4).

### 4.3. NanoString nCounter Gene Expression Assay

Tumor portion on the hematoxylin and eosin-stained FFPE tissue slides was marked by the pathologist, and total RNA was isolated from two to three FFPE tissue sections (10 μm thick) using an miRNeasy FFPE Kit (Qiagen, Hilden, Germany) according to the manufacturer’s instructions. The probe sets were custom designed and synthesized by NanoString Technologies (Seattle, WA, USA), and nCounter assays were performed according to the manufacturer’s protocol. Briefly, 500 ng of total RNA was hybridized to nCounter probe sets for 16 hours at 65 °C. Samples were then processed using an automated nCounter Sample Prep Station (NanoString Technologies, Inc., Seattle, WA, USA). Cartridges containing immobilized and aligned reporter complexes were subsequently imaged on an nCounter Digital Analyzer (NanoString Technologies, Inc.). Reporter counts were collected using the NanoString’s nSolver analysis software version 1, normalized, and analyzed. A total of eight expression probes were designed, four (5′-1 to 5′-4) proximal and four distal (3′-1 to 3′-4) to most commonly-known junction sites for *RET* fusions. An imbalance between 5′ and 3′ probe signals was indicative of the presence of a *RET* fusion transcript. We used a cutoff of three-fold for 3′/5′ ratio. Therefore, a case was considered positive for rearrangement if 3′/5′ imbalance was three-fold or more.

We used Cohen’s κ coefficient to measure agreement between RT-PCR and Nanostring method.

### 4.4. Detection of the RET/PTC1 Rearrangement in Fresh TPC1 Cell Line

Total RNA was extracted by Master Pure Complete DNA and RNA Purification Kit (Epicentre) using a fresh cell colony formed from 1000 cultured TPC1 cells (provided by Nagataki, Nakasaki University, Japan). RT-PCR was performed and the minimum number of cycles (*C*_t_ value) needed to detect the expression of *RET/PTC1* rearrangement and *GAPDH* was determined. Similarly, the number of the cells was reduced to 500, 250, 100, and 50, and RT-PCR was performed to evaluate *C*_t_ values according to cell count. The whole procedure was performed in triplicate after TPC1 cells were harvested

### 4.5. Detection of RET/PTC1 Rearrangement in Routine Air-Dried TPC1 Cell Line

To make a condition identical to that in the routine air-dried FNA preparation, cultured TPC1 cells were smeared on a slide and fixed with 95% ethanol according to the routine FNA preparation in our cytology laboratory. The fixed cells were stained by the routine Papanicolaou procedure. After the coverslips were removed from the smeared slides, the atypical cells of interest were dissected with a 26-gauge needle under the light microscope. Approximately 50, 100, 250, 500, and 1000 cells were dissected using a square micrometer under the microscope. A needle tip was carefully submerged in a tube containing extraction buffer supplied by MasterPure Complete DNA and RNA Purification Kit (Epicentre), and total RNA was extracted. RT-PCR was performed, and *C*_t_ values for the expression of *RET/PTC1* rearrangement and *GAPDH* were evaluated using 50, 100, 250, 500, and 1000 air-dried and alcohol fixed TPC1 cells, respectively. The whole procedure was performed in triplicate after TPC1 cells were harvested.

### 4.6. Correlation of RET/PTC1 Rearrangement between Routine Air-Dried FNA and Paired FFPE PTC Tissue Specimens

PTC cells from the archival FNA slides from the Department of Pathology, Konkuk University Medical Center were used. The slides that were selected were from samples aspirated from ten thyroid nodules with histopathological diagnosis of classical-type PTC. Study approval was obtained from the Institutional Review Board (KUH1210043). After the coverslips were removed from the slides, approximately 100 atypical follicular cells were dissected with a 26-gauge needle under the light microscope, and total RNA was extracted using MasterPure Complete DNA and RNA Purification Kit (Epicentre). RT-PCR was performed, and *C*_t_ values of the *RET/PTC1* rearrangement and *GAPDH* expression were evaluated. Tumor portion on the hematoxylin and eosin-stained FFPE tissue slides was marked by the pathologist, and total RNA was isolated from two-to-three FFPE tissue sections (10 μm thick) using High Pure FFPE RNA isolation kit (Roche Diagnostics). RT-PCR was performed and *C*_t_ values of the *RET/PTC1* rearrangement and *GAPDH* expression were evaluated. The *C*_t_ values defining the analysis as positive is greater than 40 cycles.

### 4.7. Detection of the RET/PTC1 Rearrangement in Resected BRAF and RAS Wild-Type PTC Cases Using FFPE Tissue Specimen

Archival thyroid neoplasm that had been surgically removed between 2010 and 2014 at Konkuk University Medical Center were blindly re-evaluated according to the 2004 World Health Organization classification of thyroid neoplasm by the two pathologists (Tae Sook Hwang, who is an endocrine pathologist, and Young Sin Ko). In case of a disagreement and to reach a consensus, another endocrine pathologist (Chan-Kwon Jung) independently reviewed the cases. Of the 600 classical PTC cases selected, 518 had *BRAF* mutation and 6 had *RAS* mutation. Finally, 76 *BRAF* and *RAS* wild-type classical PTC cases were selected. Tumor portion on the hematoxylin and eosin-stained FFPE tissue slides was marked by the pathologist, and total RNA was isolated from two-to-three FFPE tissue sections (10 μm thick) using High Pure FFPE RNA isolation kit (Roche Diagnostics). RT-PCR was performed, and *C*_t_ values of the *RET/PTC1* rearrangement and *GAPDH* expression were evaluated. The *C*_t_ value defining the analysis as positive is greater than 40 cycles.

### 4.8. Comparison Analysis of RT-PCR with the NanoString nCounter Gene Expression Assay for Detecting RET/PTC1 Rearrangement

*RET/PTC1* rearrangement status was also analyzed by the Nanostring method, using 31 cases having sufficient cancer tissue remaining for the comparative analysis.

## 5. Conclusions

*RET/PTC1* rearrangement analysis by RT-PCR using routine air-dried FNA specimen was confirmed to be technically applicable and significant population (38.2%) of the *BRAF* and R*AS* wild type PTCs harbor *RET/PTC1* rearrangement. Preoperative *RET/PTC1* rearrangement analysis in *BRAF* and *RAS* wild type indeterminate thyroid nodules would permit a formulation of unambiguous surgical plan, while foregoing the need for other less specific diagnostic test such as repeat FNA and intraoperative frozen section evaluation. An algorithmic approach is cost-effective and efficient especially in *BRAF* mutation prevalent populations.

## Figures and Tables

**Table 1:** *C*_t_ values and cell counts of *RET/PTC1* rearrangement analysis by RT-PCR using fresh cultured TPC1 cells.

Cell Number	*RET/PTC1* (*C*_t_)	*GAPDH* (*C*_t_)
1000	30.3	23.1
500	31.2	24.2
250	34.1	27.1
100	35.6	29.4
50	36.7	30.6

**Table 2:** *C*_t_ values and cell counts of *RET/PTC1* rearrangement analysis by RT-PCR using RNA extracted from routine air-dried and Papanicolaou-stained TPC1 cells.

Cell Number	*RET/PTC1* (*C*_t_)	*GAPDH* (*C*_t_)
1000	32.3	29.4
500	33.9	31.1
250	34.2	32.8
100	33.3	31.1
50	34.6	33.3

**Table 3:** Correlation of *RET/PTC1* rearrangement status between routine air-dried fine-needle aspiration (FNA) and paired formalin-fixed paraffin-embedded (FFPE) specimens.

*C*_t_ of FFPE Specimen			*C*_t_ of FNA Specimen		
Case	*RET/PTC1*	*GAPDH*	Case	*RET/PTC1*	*GAPDH*
1	26.15	27.40	1	35.05	35.86
2	26.15	29.23	2	36.41	37.54
3	26.32	28.96	3	37.03	34.79
4	40	34.67	4	50	36.74
5	24.64	28.84	5	37.66	35.86
6	31.71	29.92	6	34.26	35.47
7	24.71	26.98	7	36.62	36.78
8	50.00	28.64	8	50.00	31.69
9	50.00	27.55	9	50.00	30.34
10	50.00	29.31	10	50.00	30.01

**Table 4:** Primers and probes sequences for RT-PCR.

*RET/PTC1*	Primers and Probes Sequences
Forward primer (5′–3′)	CGC GAC CTG CGC AAA
Reverse primer (5′–3′)	CAA GTT CTT CCG AGG GAA TTC C
TaqMan Probe (5′–3′)	FAM-CCA GCG TTA CCA TCG AGG ATC CAA AGT-BHQ1
***GAPDH***	
Forward primer (5′–3′)	GTT CGA CAG TCA GCC GCA TC
Reverse primer (5′–3′)	GGA ATT TGC CAT GGG TGG A
TaqMan Probe (5′–3′)	FAM-ACC AGG CGC CCA ATA CGA CCA A-BHQ1

## Abbreviations

AUS	Atypia of undetermined significance
*C*_t_	Threshold cycle
FFPE	Formalin-fixed paraffin-embedded
FN	Follicular neoplasm;
FNA	Fine needle aspiration
FLUS	Follicular lesion of undetermined significance
FVPTC	Follicular variant of papillary thyroid carcinoma
FTC	Follicular thyroid carcinoma
NGS	Next generation sequencing
RT-PCR	Real-time reverse transcription-polymerase chain reaction
SFN	Suspicious for follicular neoplasm
PTC	Papillary thyroid carcinoma
TPC1	Thyroid papillary carcinoma 1
